# Increased monocytes and their derived indicators are associated with clinical severity of acute heart failure following acute myocardial infarction

**DOI:** 10.3389/fcvm.2025.1566635

**Published:** 2025-04-10

**Authors:** Xinlin Xiong, Minsheng An, Li Yuan, Xiaobin Long, Shen Huang

**Affiliations:** Department of Cardiology, Clinical Medical College & Affiliated Hospital of Chengdu University, Chengdu, Sichuan, China

**Keywords:** acute myocardial infarction, monocytes, monocyte-to-white blood cell ratio, monocyte-to-lymphocyte ratio, acute heart failure

## Abstract

**Objective:**

Monocytes play a significant role in the pathophysiology of acute myocardial infarction (AMI). The relationship between monocytes, their derived indicators, and the severity of acute heart failure following AMI remains unclear. Therefore, this study aims to investigate the association of monocytes and their derived indicators with clinical severity of acute heart failure in the patients with AMI.

**Methods:**

In total of 173 patients with AMI were enrolled in this retrospective study. The demographic data and relevant medical histories were obtained. Monocytes and lipid levels were measured. All patients were divided into two groups based on killip classification. Killip class III-IV was defined as acute severe heart failure, while killip class I-II was defined as acute non-severe heart failure.

**Results:**

Monocyte count, monocyte-to-white blood cell ratio, and monocyte-to-lymphocyte ratio were significantly higher in patients with acute severe heart failure compared to those with acute non-severe heart failure (*P* < 0.05). Multivariate logistic regression analysis showed that monocyte count, monocyte-to-white blood cell ratio, and monocyte-to-lymphocyte ratio were independently associated with acute severe heart failure (*P* < 0.05). Moreover, monocyte count, monocyte-to-white blood cell ratio, and monocyte-to-lymphocyte ratio were linked to NT-proBNP concentrations (*P* < 0.05). Receiver-operating characteristic curve analysis showed that monocyte count, monocyte-to-white blood cell ratio, and monocyte-to-lymphocyte ratio could identify acute severe heart failure in patients following AMI to some extent (*P* < 0.05).

**Conclusion:**

The elevation of monocyte count, monocyte-to-white blood cell ratio, and monocyte-to-lymphocyte ratio correlated with clinical severity of acute heart failure following AMI, and offered potential discriminating value for cardiogenic pulmonary edema and shock following AMI.

## Introduction

Acute myocardial infarction (AMI) is a critical medical condition. This condition represents a significant public health challenge worldwide due to its high morbidity and mortality rates (1). The burden of AMI not only impacts individual patients but also imposes substantial economic costs on healthcare systems (1, 2). The pathophysiology of AMI involves the blockage of coronary arteries due to plaque rupture and subsequent thrombus formation. This blockage prevents oxygen-rich blood from reaching the myocardial tissue, resulting in cellular injury and tissue damage or necrosis. Despite advances in medical technology and therapeutic strategies, AMI remains associated with severe complications, such as arrhythmias, cardiac arrest, and acute severe heart failure (3). Acute severe heart failure, in particular, is a major concern for patients who survive an initial myocardial infarction. AMI with accompanying heart failure is associated with a worse prognosis compared to AMI without heart failure (1, 4). Acute severe heart failure arises from an inability to maintain cardiac output due to the loss of contractile function in the damaged myocardial region and subsequent decompensation. These pathological changes result in a rapid decline in cardiac function, leading to pulmonary edema and cardiogenic shock.

In recent years, there has been a growing understanding of the crucial role that the immune system, particularly monocytes, and lymphocytes, play in the pathophysiology of AMI (5–11). Moreover, monocytes and their derived indicators have been investigated for their potential to predict the presence of major adverse cardiovascular events in patients with AMI in clinical practice (12). The deceased patients with AMI exhibit higher monocyte count and elevated monocyte-to-lymphocyte ratio compared to survived patients, with these indicators also correlating with cardiovascular mortality, even after adjusting for relevant covariates (13). However, the relationship between monocytes, and their derived indicators and clinical severity of acute heart failure following AMI remains unclear. Therefore, this study aims to investigate the relationship between these inflammatory indicators and acute severe heart failure in the patients with AMI.

## Patients and methods

### Study participants

In total of 173 patients with AMI were enrolled in this retrospective study from January 2021 to May 2022. The diagnostic criteria for AMI were based on the fourth universal definition of AMI (14), which included: a rise and/or fall of cardiac troponin values with at least one value exceeding the 99th percentile upper reference limit, along with at least one of the following: ischaemic symptom, electrocardiogram changes, progressive pathological Q waves, recent loss of viable myocardial tissue confirmed by imaging, coronary thrombus found by angiography (14). The inclusion criteria included non-ST elevation myocardial infarction, and ST-elevation myocardial infarction. All patients with AMI underwent coronary angiography, which revealed ≥50% stenosis in one or more of any major epicardial coronary artery. The exclusion criteria were as follows: myocardial infarction due to other causes such as spasm, dissection of aorta, embolism, severe liver or renal dysfunction, severe infection, severe anemia, malignant tumour, or active autoimmune disease. This study was approved by the Ethics Committee of the Clinical Medical College and Affiliated Hospital of Chengdu University. The study was conducted in accordance with the Declaration of Helsinki. Written informed consent to participate in the study was not required from the participants in accordance with the local legislation and institutional requirements.

### Data collection

The clinical data were obtained. We collected information on age, gender, hypertension, diabetes mellitus, smoking status, and blood lipid levels. The blood lipid parameters included low-density lipoprotein cholesterol, triglycerides, high-density lipoprotein cholesterol, total cholesterol. Low-density lipoprotein cholesterol and high-density lipoprotein cholesterol were determined via an automatic biochemical analyzer with the application of the direct method. Total cholesterol and triglycerides were gauged by an automatic biochemical analyzer employing the oxidase assay.

### Blood test data

Blood samples were collected from patients on admission. During blood specimen collection, the choice of collection tubes with anticoagulants varies depending on the measurement indicators. For measuring blood lipids, no anticoagulant tube is needed; for measuring blood routine parameters, an EDTA anticoagulant tube is required; and for measuring N-terminal probrain natriuretic peptide (NT-proBNP), a heparin anticoagulant tube is needed. White blood cell count, monocyte count, neutrophil count, lymphocyte count, and NT-proBP concentrations were measured in clinical laboratory. Derived ratios were calculated as follows: neutrohil-to-white blood cell ratio = (neutrophil count/white blood cell count) × 100%, neutrophil-to-lymphocyte ratio = (neutrophil count/lymphocyte count) × 100%, monocyte-to-white blood cell ratio = (monocyte count/white blood cell count) × 100%, monocyte-to-lymphocyte ratio = (monocyte count/lymphocyte count) × 100%, lymphocyte-to-white blood cell ratio = (lymphocyte count/white blood cell count) × 100%. The monocyte-derived indicators included monocyte-to-white blood cell ratio and monocyte-to-lymphocyte ratio.

### The definition of acute severe heart failure

Acute severe heart failure following AMI in hospital was defined as killip classification class III–IV, which encompassed cardiogenic pulmonary edema and shock, and was associated with poor prognosis (1, 15). These patients with acute non-severe heart failure following AMI were classified as killip class I–II, characterized by rales in <50% of the lung area or the absence of pulmonary rales.

### Statistical analysis

The normality of the distribution is analyzed using the Shapiro–Wilk test. Continuous normally distributed variables were expressed as mean and standard deviation (SD), and continuous non-normally distributed variables were displayed as median and interquartile range (IQR). Categorical variables were presented as frequency and percentage. Continuous variables with normal distribution were compared using the the Student's t-test. Continuous variables with non-normally distributed data were analyzed by the the Mann–Whitney U-test. The Chi-squared (*χ*^2^) test was used to compare categorical variables between groups. Receiver-operating characteristic (ROC) curves were constructed to calculate the cut-off values for monocytes and their derived indicators to discriminate acute severe heart failure, and to determine the area under the curve (AUC) with 95% confidence interval (CI). Spearman's rank correlation analysis was conducted to investigate the associations between monocytes and their derived indicators with NT-proBNP levels. Univariate and multivariable logistic regression analyses were used to assess the association of monocytes and their derived indicators with acute severe heart failure, odds ratio (OR) and 95% CI were calculated. Statistical analyses were performed using SPSS 26.0, *P* < 0.05 was considered statistically significant.

## Result

### Baseline characteristics of the patients with AMI

The median age of AMI patients was 62 years, with an interquartile range (25th–75th percentile) of 51.00–72.50 years. Among these participants, 79.2% were male, 48.6% had hypertension, 21.4% had diabetes mellitus, and 65.3% were smokers. The baseline characteristics of the patients were shown in Table 1. Patients with acute severe heart failure were more likely to be older than those with acute non-severe heart failure (*P* < 0.05). The prevalence of hypertension was higher in the group with acute severe heart failure than in the group with acute non-severe heart failure (*P* < 0.05). High density lipoprotein cholesterol was significantly higher in the group with acute sever heart failure than in the group with acute non-severe heart failure (*P* < 0.05). The NT-proBNP levels were markedly increased in the group with acute sever heart failure compared to the group with acute non-severe heart failure (*P* < 0.05). The prevalence of diabetes mellitus, smoking, and the proportion of male were similar between the groups. There were no difference in the levels of triglycerides, total cholesterol, and low density lipoprotein cholesterol (*P* > 0.05).

**Table 1:** Baseline characteristics of patients between acute severe heart failure and acute non-severe heart failure.

Variables	Group with acute non-severe heart failure (*n* = 95)	Group with acute severe heart failure (*n* = 78)	*P*
Age (years)	58.95 ± 12.93	64.15 ± 13.40	0.010
Male, *n* (%)	76 (80.0)	61 (78.2)	0.772
Hypertension, *n* (%)	38 (40.0)	46 (59.0)	0.013
Diabetes mellitus, *n* (%)	20 (21.1)	17 (21.8）	0.906
Smoking, *n* (%)	64 (67.4)	49 (62.8)	0.532
NSTEMI/STEMI	22/73	22/56	0.448
Triglycerides (mmol/L) [<1.7 mmol/L]^a^	1.22 (0.70, 2.06)	1.26 (0.84, 1.79)	0.728
TC (mmol/L) [0–5.18 mmol/L]^a^	4.43 ± 0.91	4.48 ± 0.97	0.739
HDL-C (mmol/L) [≥1.00 mmol/L]^a^	0.97 (0.85, 1.08)	1.02 (0.89, 1.22)	0.034
LDL-C (mmol/L) [<3.4 mmol/L]^a^	3.05 ± 0.87	2.97 ± 0.88	0.510
NT-proBNP (pg/ml) [0–300pg/ml]^a^	377.55 (107.38, 1,323.50)	1002.00 (212.00, 2,823.00)	<0.001

^a^ Normal range for the reported laboratory parameters. The values are presented as median (25th–75th percentiles) for non-normally distributed variables, mean (standard deviation) for normally distributed data, *n* (%) for categorical variables. NT-proBNP and lipids were available from 171 and 168 participants, respectively. NSTEMI, non-ST elevation myocardial infarction; STEMI, ST elevation myocardial infarction; HDL-C, high density lipoprotein cholesterol; LDL-C, low density lipoprotein cholesterol; TC, total cholesterol; NT-proBNP, N-terminal proBNP. acute non-severe heart failure, defined as killip class I–II; acute severe heart failure, defined as killip class III–IV.

### Elevated monocyte count and monocyte-derived indicators in patients with acute severe heart failure following AMI

We compared monocyte count, neutrophil count, lymphocyte count, monocyte-to-white blood cell ratio, monocyte-to-lymphocyte ratio, and lymphocyte-to-white blood cell ratio as well as neutrophil-to-white blood cell ratio and neutrophil-to-lymphocyte ratio between the patients with acute severe heart failure and with acute non-severe heart failure. As shown in Table 2, monocyte count was significantly higher in the patients with acute severe heart failure than those with acute non-severe heart failure. Similarly, monocyte-to-white blood cell ratio and monocyte-to-lymphocyte ratio were also higher in patients with acute severe heart failure compared to those with acute non-severe heart failure (Table 2). However, neutrophil count, lymphocyte count, lymphocyte-to-white blood cell ratio, neutrophil-to-white blood cell ratio, and neutrophil-to-lymphocyte ratio were comparable between the two groups (Table 2).

**Table 2:** The laboratory parameters of patients between acute severe heart failure and acute non-severe heart failure.

Variables	Group with acute non-severe heart failure (*n* = 95)	Group with acute severe heart failure (*n* = 78)	*P*
Monocytes [0.1–0.6]*10^9^/L^a^	0.58 (0.44, 0.74)	0.72 (0.53, 0.94)	0.004
Lymphocytes [1.1–3.2]*10^9^/L^a^	1.31 (0.93, 1.79)	1.22 (0.86, 1.63)	0.306
Neutrophils [1.8–6.3]*10^9^/L^a^	8.15 (6.74, 10.58)	8.77 (5.93, 10.80)	0.818
Monocyte-to-white blood cell ratio (%)	5.90 ± 2.41	7.06 ± 2.57	0.003
Lymphocyte-to-white blood cell ratio (%)	13.4 (8.60, 19.20)	12.50 (7.88, 18.25)	0.377
Neutrophil-to-white blood cell ratio (%)	79.10 (71.9, 86.50)	79.90 (72.48, 85.75)	0.842
Monocyte-to-lymphocyte ratio (%)	42.00 (30.00, 58.00)	50.50 (37.75, 84.00)	0.001
Neutrophil-to-lymphocyte ratio (%)	589.00 (374.00, 1,022.00)	631.00 (398.25, 1,075.25)	0.479

^a^ Normal range for the reported laboratory parameters. The values are presented as median (25th–75th percentiles) for non-normally distributed variables, mean (standard deviation) for normally distributed data. acute non-severe heart failure, defined as killip class I–II; acute severe heart failure, defined as killip class III–IV.

### Association of monocytes and their derived indicators with acute severe heart failure

We carried out logistic regression analysis to investigate the association between monocytes and their derived indicators and acute severe heart failure. Univariate logistic regression analysis showed that monocyte count, monocyte-to-white blood cell ratio, and monocyte-to-lymphocyte ratio were significantly associated with acute severe heart failure. The OR values were 3.518 (95% CI = 1.331–9.298, *P* < 0.05), 1.208 (95% CI = 1.064–1.371, *P* < 0.01), 7.244 (95% CI = 2.288–22.934, *P* < 0.01), respectivley. After adjusting for covariant, including age, gender, hypertension, diabetes mellitus, smoking status, low-density lipoprotein cholesterol, triglycerides, high-density lipoprotein cholesterol, total cholesterol, multivariate logistic regression analysis using variables selected by the forward likelihood ratio method was implemented, the results showed that monocyte count was independently associated with acute severe heart failure (OR = 4.480, 95% CI = 1.625–12.354, *P* < 0.01). In addition, a significant association between monocyte-to-white blood cell ratio and acute severe heart failure following AMI was observed, with an adjusted OR value of 1.232 (95% CI = 1.078–1.409, *P* < 0.01). Furthermore, the relationship between monocyte-to-lymphocyte ratio and acute severe heart failure remained statistically significant (OR=6.592, 95% CI = 2.080–20.891, *P* < 0.01).

### The association of monocytes and their derived indicators with NT-proBNP

**Figure 1:**
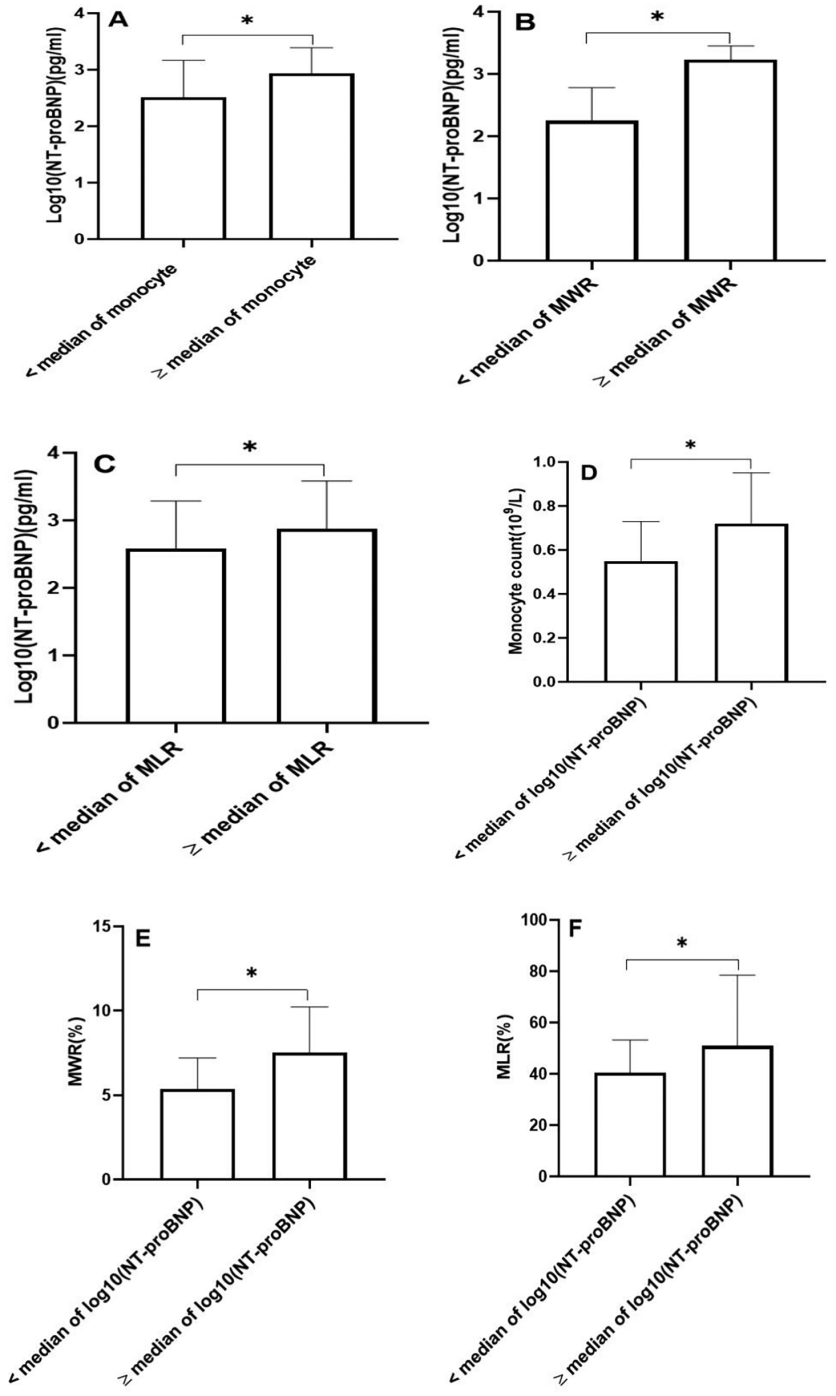
The N-terminal probrain natriuretic peptide (NT-proBNP) concentrations, monocyte count, monocyte-to-white blood cell ratio (MWR), and monocyte-to-lymphocyte ratio (MLR). **(A)** The concentrations of NT-proBNP according to the median of monocyte count. **(B)** The concentrations of NT-proBNP according to the median of MWR. **(C)** The concentrations of NT-proBNP according to the median of MLR. **(D)** The monocyte count according to the median of log10(NT-proBNP). **(E)** The MWR according to the median of log10(NT-proBNP). **(F)** The MLR according to the median of log10(NT-proBNP). (*P < 0.01). NT-proBNP values were logarithmically transformed for statistical analysis.

We examined the relationship between monocytes and their derived indicators and NT-proBNP. NT-proBNP values were logarithmically transformed for statistical analysis. The patients with AMI were classified into two groups according to the median of monocyte count. We found that NT-proBNP concentrations were significantly higher in the group with monocyte count ≥ median value compared to the group with monocyte count < median value (Figure 1A). Similarly, we also divided the patients into two groups according to the median of monocyte-to-white blood cell ratio and monocyte-to-lymphocyte ratio. The group with monocyte-to-white blood cell ratio ≥ median value demonstrated higher NT-proBNP concentrations (Figure 1B). In addition, patients with high monocyte-to-lymphocyte ratio exhibited significantly higher NT-proBNP levels compared to those with low monocyte-to-lymphocyte ratio (Figure 1C). In order to further confirm the relationship between monocytes, monocyte-derived indicators, and NT-proBNP, we also classified these patietns into two groups according to the median of the logarithm of NT-proBNP values. Monocyte count was found to be elevated in the group with a logarithm of NT-proBNP ≥ median compared to the group with a logarithm of NT-proBNP < median (Figure 1D). Moreover, we noted a significant rise in monocyte-to-white blood cell ratio and monocyte-to-lymphocyte ratio in the group with a logarithm of NT-proBNP at or above the median, relative to the group with a logarithm of NT-proBNP below the median (Figures 1E,F). We further conducted a correlation analysis using spearman's rank correlation test to evaluate the association of monocytes and their derived indicators with NT-proBNP levels. The results demonstrated significant positive correlations, with coefficients of 0.279 for monocyte count, 0.492 for the monocyte-to-white blood cell ratio, and 0.265 for the monocyte-to-lymphocyte ratio, all exhibiting statistically significant *P*-values < 0.01.

### Value of monocytes and their derived indicators to discriminate the patients with acute severe heart failure

**Figure 2:**
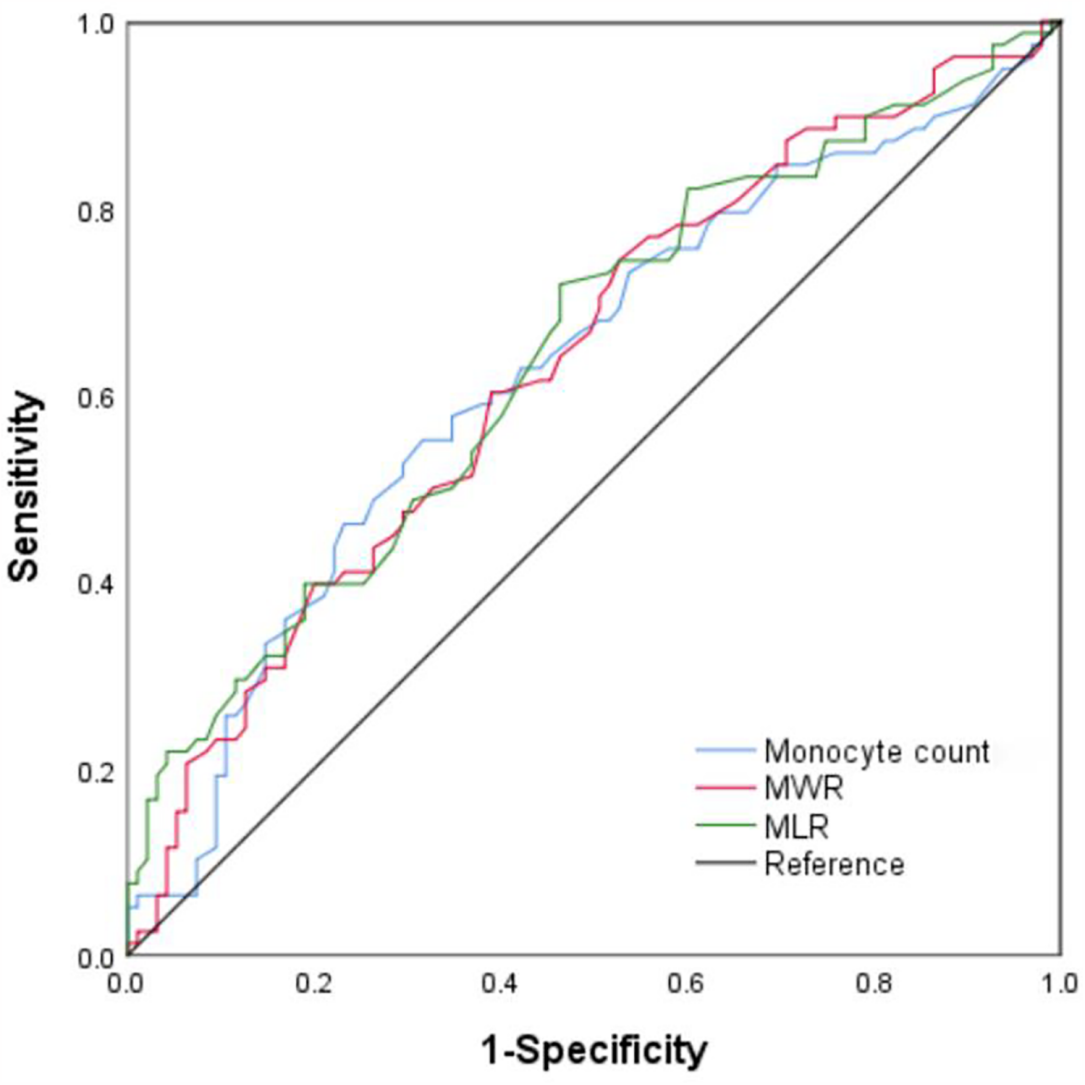
ROC curve analysis of monocyte count, monocyte-to-white blood cell ratio (MWR), monocyte-to-lymphocyte ratio (MLR) for acute severe heart failure. The AUC values for monocyte count, MWR, and MLR are 0.628, 0.634, and 0.643, respectively. (All *P* < 0.01). ROC, receiver-operating characteristic; AUC, area under the curve.

We performed the ROC curve analysis to assess the AUC and optimal cut-off value for monocytes and their derived indicators in discriminating acute severe heart failure in patients following AMI (Figure 2). The area under the curve (AUC) for the monocyte count to distinguish acute severe heart failure was 0.628 [95% confidence interval (CI) = 0.544–0.712, *P* < 0.01], with a sensitivity and a specificity of 55.1% and 68.4%. The optimal cut-off value for monocyte count as a discriminative marker of the patients with acute severe heart failure was 0.695 × 109/L. The AUC for monocyte-to-white blood cell ratio was 0.634 (95% CI = 0.551–0.717, *P* < 0.01), and the optimal diagnostic cut-off point was 5.35%, with a sensitivity of 74.4% and a specificity of 47.4%. The AUC for monocyte-to-lymphocyte ratio was 0.643 (95% CI = 0.560–0.726, *P* < 0.01), and the optimal diagnostic cut-off point was 42.5%, with a sensitivity of 71.8% and a specificity of 53.7%.

## Discussion

The findings of our study demonstrated that monocyte count, as well as monocyte-to-white blood cell ratio and monocyte-to-lymphocyte ratio, were significantly elevated in patients with AMI complicated by acute severe heart failure. This elevation not only correlated with the severity of acute heart failure but also serves as a potential differentiator in the clinical assessment of AMI patients in the hospital. However, these inflammtory indicators including neutrophil count, lymphocyte count, neutrophil-to-white blood cell ratio, neutrophil-to-lymphocyte ratio, and lymphocyte-to-white blood cell ratio were not altered. The pathophysiological implications of these findings warrant further exploration.

Atherosclerosis was widely recognized as a chronic inflammatory disorder rather than simply a lipid-driven disease. Its pathogenesis involves complex interactions between lipids, endothelial cells, smooth muscle cells, and various immune cells (16–18). Monocytes were the important component of the innate immune system, and a type of white blood cell. Recent literature highlights the role of monocytes and their derived indicators in coronary artery disease. A study had suggested that the elevated monocyte was linked to coronary artery stenosis in stable coronary artery disease patients (19), and monocyte-to-lymphocyte ratio was a predictive factor for coronary artery disease (20). Monocyte-to-lymphocyte ratio correlated with the subclinical lesion of coronary arteries with coronary artery calcification scores of 100 or above (21). The monocyte-to-lymphocyte ratio was independently linked to the severity of coronary artery stenosis (22). Another cross-sectional study showed that monocyte-to-lymphocyte ratio could identify patients with coronary artery disease combined with type 2 diabetes mellitus (23). Monocyte-to-lymphocyte ratio was positively associated with burden of coronary lesion in patients with non-ST-elevation myocardial infarction (24). These studies mentioned above indicated that monocytes and their derived indicators were associated with occurence and progression of coronary artery disease.

Furthermore, the monocyte-to-lymphocyte ratio had been identified as a promising biomarker for risk stratification of coronary artery disease. The all-cause mortality, cardiac mortality, and major adverse cardiovascular and cerebrovascular events were significantly higher in patients with coronary artery disease after percutaneous coronary intervention when monocyte-to-lymphocyte ratio was high (25). A study by Jiang R et al. reported that increased monocyte-to-lymphocyte ratio was linked to both cardiovascular and all-cause mortalities according to analysis of Kaplan–Meier and multivariate cox regression in coronary artery disease patients with low-density lipoprotein cholesterol concentration below 1.4 mmol/L, suggesting an association of monocyte-to-lymphocyte ratio with the risk of residual inflammation (26). The study from a retrospective cohort analysis found that monocyte-to-lymphocyte ratio also correlated with 1-year mortality in patients with AMI in critical condition (27). Moreover, monocyte-to-lymphocyte ratio was positively associated with long-term major adverse cardiac event in patients with non-ST-elevation myocardial infarction (24). These studies have demonstrated that the monocyte-to-lymphocyte ratio was a predictor of poor outcomes in patients with AMI.

In addition, monocytes might also play a significant part in the development of AMI (10, 11, 28). Monocytes are heterogeneous, with different subsets having distinct functions (12, 28). Monocytes are integral components of the immune response and are activated in the setting of myocardial ischemia (9). Upon injury, monocytes migrate to the site of infarction, where they play dual roles in both inflammation and tissue repair. This recruitment process is mediated by a complex interplay of cytokines and chemokines, which are released by damaged myocardial tissue (9, 29, 30). The presence of activated monocyte has been linked to enhanced release of pro-inflammatory cytokines, such as TNF-α and IL-6 (11), which contributed to myocardial damage and adverse remodeling. Monocytes could differentiate into macrophages, which are involved in phagocytosis of necrotic debris (31, 32). The dysregulation of monocyte function has been implicated in the rapid progression from AMI alone to a state associated with severe acute heart failure (31). In our study, we found that monocytes and their derived indicators (monocyte-to-white blood cell ratio, monocyte-to-lymphocyte ratio) were significantly elevated in patients experiencing acute severe heart failure compared to those with acute non-severe heart failure, indicating that these markers were associated with the severity of acute heart failure following myocardial infarction. This may reflect an exaggerated pro-inflammatory state, with monocytes potentially driving the inflammatory process at the expense of the anti-inflammatory and regulatory functions of other immune cells. Similar to acute severe heart failure, cardiac rupture, which indicated severe myocardial damage, represented a critical and frequently life-threatening complication of AMI, Dai K et al. found that increased monocyte-to-lymphocyte ratio could discriminate cardiac rupture in patients with AMI, suggesting that elevated monocyte-to-lymphocyte ratio might contribute to severe myocardial necrosis (33). Lu Q et al. also revealed that inflammation was independently associated with cardiac rupture (34). In addition, our study also found that monocytes and their derived indicators could identify acute severe heart failure in these patients experiencing AMI, indicating that monocyte count, monocyte-to-white blood cell ratio, and monocyte-to-lymphocyte ratio were important biomakers to predict acute severe heart failure in patients with AMI. NT-ProBNP has been extensively utilized in the patients with heart failure. Literature reported that NT-ProBNP was associated with severity of heart failure (35). Consistent with the literature reports, our study also revealed that NT-ProBNP was significantly increased in patients with acute severe heart failure compared to the patients with acute non-severe heart failure. Therefore, in addition to being used as a biomaker for diagnosis of heart failure (36), NT-ProBNP was also a sensitive biomarker for assessing severity of heart failure. In our study, we found that the NT-ProBNP concentrations in patients with high level of monocytes and their derived indicators were obviously elevated compared to the patients with low level of monocytes and their derived indicators, further supporting our finding that monocytes and their derived indicators were associated with the severity of heart failure. Moreover, the ability to differentiate patients with AMI who develop acute severe heart failure from those who do not was particularly important for clinical management. Literature has shown that early identification of acute severe heart failure in the context of AMI could lead to timely interventions that improve survival (1). We demonstrated that monocytes and their derived indicators exhibited some abilities to recognize acute severe heart failure by ROC curve analysis. However, unlike NT-ProBNP, monocytes served as an inflammatory marker, potentially reflecting the inflammatory burden associated with AMI complicated by acute heart failure. An increased inflammatory load might indicate more severe acute heart failure following AMI. Our findings suggested that monitoring monocytes and their dreived indicators (monocyte count, monocyte-to-white blood cell ratio, monocyte-to-lymphocyte ratio) could be instrumental in assessing the inflammatory burden in patients presenting with AMI. By identifying monocyte-related biomarkers, we could enhance clinical decision-making and optimize treatment strategies for this vulnerable population.

While our study presented valuable insights, it was not without limitations. Several limitations must be acknowledged in present study. Firstly, due to the retrospective study, some variables that are difficult to obtain, such as coronary artery lesion characteristics, body mass index, and other potential factors, were not included, which may introduce bias into the results. Therefore, we must exercise caution when interpreting these findings. Moreover, we found the neutrophils and lymphocytes were similar in both these patients with acute severe heart failure and those with acute non-severe heart failure, however, some of their subsets might be different, further study was needed. Another important consideration is the need for further exploration of potential therapeutic strategies that target monocyte activity. Such interventions could offer novel approaches for improving patient outcomes in both acute myocardial infarction and heart failure. Furthermore, a more in-depth investigation into how monocyte dynamics interacted with other immune cell populations in the context of myocardial injury would provide crucial insights into the pathophysiology of heart failure, potentially unveiling new targets for treatment.

## Conclusion

In summary, our study highlighted the significant role of monocytes and their derived indicators in the context of AMI and acute heart failure. The elevation of monocyte count, monocyte-to-white blood cell ratio, and monocyte-to-lymphocyte ratio correlated with clinical severity of acute heart faiure following AMI, and offered potential discriminating value for cardiogenic pulmonary edema and shock following AMI. These findings underscore the need for further investigation into the pathophysiological mechanisms and the potential for novel therapeutic approaches targeting inflammation in acute heart failure following AMI.

## Data Availability

The original contributions presented in the study are included in the article/Supplementary Material, further inquiries can be directed to the corresponding author.
